# First-Line Treatment of Metastatic Clear Cell Renal Cell Carcinoma: What Are the Most Appropriate Combination Therapies?

**DOI:** 10.3390/cancers13215548

**Published:** 2021-11-05

**Authors:** Yann-Alexandre Vano, Sylvain Ladoire, Réza Elaidi, Slimane Dermeche, Jean-Christophe Eymard, Sabrina Falkowski, Marine Gross-Goupil, Gabriel Malouf, Bérangère Narciso, Christophe Sajous, Sophie Tartas, Eric Voog, Alain Ravaud

**Affiliations:** 1 Georges Pompidou European Hospital, 75015 Paris, France; 2 Georges François Leclerc Centre, 21000 Dijon, France; sladoire@cgfl.fr; 3 Association for the Research of Innovative Therapeutics in Cancerology (ARTIC), 75015 Paris, France; relaidi@gmail.com; 4 Paoli Calmettes Institute, 13009 Marseille, France; DERMECHES@ipc.unicancer.fr; 5 Jean Godinot Institute, 51100 Reims, France; jc.eymard@reims.unicancer.fr; 6 Limoges Polyclinic, 87000 Limoges, France; s.falkowski@polyclinique-limoges.fr; 7 Saint André Hospital, Bordeaux University Hospital, 33000 Bordeaux, France; marine.gross-goupil@chu-bordeaux.fr; 8 Institute of Cancerology of Strasbourg (ICANS), 67200 Strasbourg, France; maloufg@igbmc.fr; 9 Tours University Hospital, 37000 Tours, France; berengere.narciso@univ-tours.fr; 10 Lyon Civil Hospices Institute of Cancerology, Pierre Bénite, 69002 Lyon, France; christophe.sajous@chu-lyon.fr (C.S.); sophie.tartas@chu-lyon.fr (S.T.); 11 Victor Hugo Clinic, Inter-Regional Institute of Cancerology, 72000 Le Mans, France; e.voog@ilcgroupe.fr; 12 Bordeaux University Hospital, 33000 Bordeaux, France; alain.ravaud@chu-bordeaux.fr

**Keywords:** metastatic clear cell renal cell carcinoma, first-line treatment, immunotherapy, tyrosine kinase inhibitors, combinations

## Abstract

**Simple Summary:**

First-line treatment options for metastatic clear cell renal cell carcinoma have significantly increased. The current recommended therapeutic strategy is based on a combination, but monotherapy remains an alternative. However, the choice of the type of combination, i.e., dual immunotherapy or immunotherapy combined with an antiangiogenic drug, has not been clearly standardized. A strategy based on the International Metastatic Database Consortium (IMDC) classification is currently recommended with pembrolizumab + axitinib, cabozantinib + nivolumab, and lenvatinib + pembrolizumab (for all patients) or nivolumab + ipilimumab (for patients with intermediate or poor risk), which are the first-line treatment standards of care. This review summarizes all recent data from the main combinations evaluated in first-line treatment and discusses the choice of drugs according to the patient’s profile and the benefit/risk balances of each combination.

**Abstract:**

The development of antiangiogenic treatments, followed by immune checkpoint inhibitors (ICI), has significantly changed the management of metastatic clear cell renal cell cancer. Several phase III trials show the superiority of combination therapy, dual immunotherapy (ICI-ICI) or ICI plus tyrosine kinase inhibitors (TKI) of the vascular endothelium growth factor (VEGF) over sunitinib monotherapy. The question is therefore what is the best combination for a given patient? A strategy based on the International Metastatic Database Consortium (IMDC) classification is currently recommended with pembrolizumab + axitinib, cabozantinib + nivolumab, and lenvatinib + pembrolizumab (for all patients) or nivolumab + ipilimumab (for patients with intermediate or poor risk), which are the first-line treatment standards of care. However, several issues remain unresolved and require further investigation, such as the PD-L1 status, the relevance of possible options based on the patient’s profile, and consideration of second-line and subsequent treatments.

## 1. Introduction

Clear cell renal cell carcinoma (ccRCC) used to be associated with a very poor prognosis when diagnosed at an advanced stage. The last 15 years have provided dramatic improvements in this field, thanks to the development of vascular endothelial growth factor (VEGF) tyrosine kinase inhibitors (TKI) followed by immune checkpoint inhibitors (ICIs) [1,2]. ICIs are monoclonal antibodies directed against immune checkpoints and enable the reversal of tumor-induced immunosuppression. Currently, the anti-checkpoint agents used in oncology target inhibitory receptors present on the surface of lymphocytes such as programmed cell death 1 (PD-1) and cytotoxic T lymphocyte–associated protein 4 (CTLA-4) or their ligands (PD-L1, programmed cell death ligand 1) [3,4]. Combining therapies to further improve survival and response rates has been tested in large phase III randomized trials, in particular CheckMate-214 (nivolumab (PD-1) + ipilimumab (CTLA-4) vs. sunitinib (TKI)), JAVELIN Renal 101 (axitinib (TKI) + avelumab (PD-L1), vs. sunitinib), KEYNOTE-426 (axitinib + pembrolizumab (PD-1) vs. sunitinib), CheckMate 9ER (nivolumab + ipilimumab vs. sunitinib) and CLEAR (lenvatinib (TKI) + pembrolizumab) [5,6,7,8,9,10,11,12]. These trials were positive, showing the superiority of the combination, i.e., dual immunotherapy (ICI-ICI) or ICI plus TKI (ICI-TKI) over sunitinib monotherapy. A recent meta-analysis including these trials confirms that immune-based combinations are more effective than sunitinib monotherapy with a three-fold increase in the complete response rate [4]. According to the recently updated European guidelines, lenvatinib + pembrolizumab joins other VEGFR+PD-1 inhibitor-targeted combinations (axitinib + pembrolizumab or cabozantinib + nivolumab) to be recommended for first-line treatment of advanced ccRCC irrespective of International Metastatic RCC Database Consortium (IMDC) risk groups. Ipilimumab + nivolumab also continues to be recommended for first-line treatment of IMDC intermediate- and poor-risk (I/P) patients [13,14]. One of the most critical emerging questions now is how to select the best option for a given patient? A recent article suggested treatment algorithms for first-line treatment in metastatic ccRCC (mccRCC) with a wide spectrum of treatment recommendations based on multiple decision criteria demonstrated. Significant inter-expert variations were observed [15]. Herein, we review recent data and discuss how, for a given patient, the best strategy should be chosen. Our approach integrates data available in routine clinical practice, such as effectiveness data, IMDC groups, PD-L1 status, tolerability of treatments and perspectives of treatment sequence. 

## 2. Overview of Studies in First-Line Metastasis

**Figure 1:**
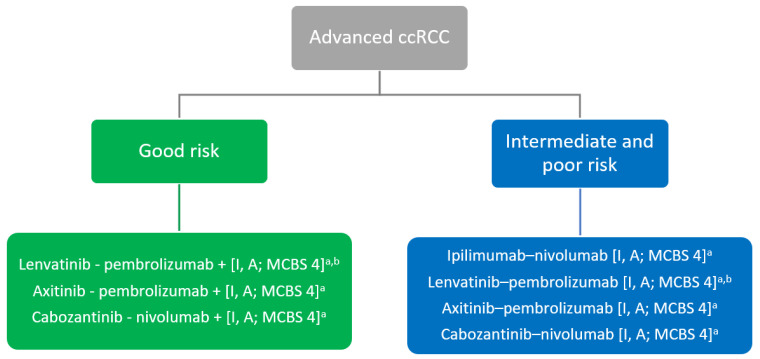
ESMO Clinical Practice Guideline update: Systemic first-line treatment of clear cell renal cell carcinoma (ccRCC) [14]. ccRCC, clear cell renal cell cancer; EMA, European Medicines Agency; ESMO-MCBS, European Society for Medical Oncology-Magnitude of Clinical Benefit Scale; FDA, Food and Drug Administration; IMDC, International Metastatic RCC Database Consortium; MCBS, ESMO-Magnitude of Clinical Scale; VEGFR, vascular endothelial growth factor receptor. a ESMO-MCBS v1.1 score for new therapy/indication approved by the EMA or FDA. The score has been calculated by the ESMO-MCBS Working Group and validated by the ESMO Guidelines Committee; b FDA approved; not currently EMA approved.

Today, the European Society for Medical Oncology (ESMO) recommends dual immunotherapy (ICI-ICI) or a combination of immunotherapy and antiangiogenics (ICI-TKI) for patients with mccRCC. Dual immunotherapy is recommended only for patients with an intermediate or poor risk tumor, which constitutes approximately 80% of patients with advanced ccRCC (Figure 1) [13,14].

This combination improves survival outcome in these patients with mccRCC. The CheckMate-214 study comparing nivolumab + ipilimumab (NIVO + IPI) to sunitinib (SUN) showed results in favor of the combination, which was confirmed by updated results over four years [5,6]. Overall survival (OS) (hazard ratio (HR); 95% confidence interval (CI)) remained superior with NIVO + IPI compared with SUN in the intention-to-treat (ITT) population (0.69; 0.59 to 0.81) and particularly in patients with I/P disease (0.65; 0.54 to 0.78). Four-year progression-free survival (PFS) rates were 31.0% vs. 17.3% (ITT) and 32.7% vs. 12.3% (I/P) in the NIVO + IPI group vs. SUN. The objective response rate (ORR) remained higher with NIVO + IPI vs. SUN in the ITT population (39.1% vs. 32.4%) and in the I/P risk group (41.9% vs. 26.8%). Similarly, the complete response rate (CR) was 10.7% vs. 2.6% in the ITT population and 10.4% vs. 1.4% in the I/P risk population for the NIVO + IPI groups vs. SUN, respectively.

The first major trial for the ICI plus VEGFR TKI with axitinib combination was KEYNOTE-426. The first results at 14 months and then at 30 months were clearly in favor of the pembrolizumab + axitinib (PEMBRO + AXI) combination [9,10]. The 42.8-month update confirmed the superiority over all endpoints in the ITT population: median OS of 45.7 vs. 40.1 months (HR 0.73 [95% CI: 0.60, 0.88], *p* < 0.001), median PFS of 15.7 vs. 11.1 months (HR 0.68 [0.58–0.80], *p* < 0.0001) and ORR 60% (10% CR) vs. 40% (3.5% CR) (*p* < 0.0001), respectively [16]. These results confirmed the status of PEMBRO + AXI as a first-line treatment standard for all patients according to the latest European recommendations [13,14]. Another immunotherapy combination trial—the JAVELIN Renal 101 trial—reported, with 13 months of follow-up, superior PFS of avelumab + axitinib (AVE + AXI) vs. SUN, whether in patients with PD-L1 positive (PD-L1^+^) tumors (HR 0.61; *p* < 0.0001; 13.8 vs. 7.2 months) or in the overall population (HR 0.69; *p* < 0.0001; 13.8 vs. 8.4 months). This combination did not appear in the recommendations due to a lack of OS benefit [7,8]. A third interim analysis over more than two years confirmed these data, with a non-statistically significant OS benefit and a PFS of 13.9 vs. 8.5 months (HR 0.67; *p* < 0.0001) [17]. More recently, the CheckMate 9ER study evaluated the cabozantinib and nivolumab (CABO + NIVO) combination and showed an OS benefit compared with SUN monotherapy (HR 0.6; 95% CI: 0.4–0.49; *p* = 0.001) and PFS (16.6 vs. 8.3 months; HR 0.51; 95% CI: 0.41–0.64; *p* < 0.0001) with an ORR of 55.7%, including 8% of CR [11]. With an 18-month follow-up, this trial was largely positive for survival and response rates. Only 6% of patients were progressive from the outset, and this combination was therefore also promising. The last combination of interest was lenvatinib + pembrolizumab (LENVA + PEMBRO) compared with SUN monotherapy in the phase III CLEAR study in 1069 treatment-naïve patients with mccRCC [12]. With a median follow-up of 27 months, this study was clearly positive for its primary endpoint with a median PFS of 23.9 months (20.8–27.7) in the LENVA + PEMBRO arm versus 9.2 months (6.0–11.0) in the SUN arm (HR 0.39; 0.32–0.49; *p* < 0.001). PFS was improved regardless of the IMDC subgroup or sarcomatoid contingent. Lenvatinib + everolimus also met the primary endpoint, with median PFS of 14.7 months versus 9.2 months for SUN, representing a 35% improvement in favor of this combination. The LENVA + PEMBRO combination significantly improved OS compared to SUN (HR 0.66; 0.49–0.69; *p* = 0.005) with a particularly marked benefit in IMDC poor risk group (HR 0.30). The ORR in the LENVA + PEMBRO arm was 71%, including 16% of CR. Only 5.4% of patients experienced immediate progression following the introduction of LENVA + PEMBRO. It should be noted that in this study a high proportion of patients had a good prognosis, with a spontaneously more favorable history. Nevertheless, this combination provided the longest PFS or OS durations ever reported in a pivotal phase III trial (Table 1).

## 3. Pending Questions and Impact on Clinical Practice

### 3.1. Comparisons of Combinations

Given the number of effective first-line treatment options and the absence of direct comparison studies, the major question is which combination to prescribe to which patients? A recent meta-analysis indirectly compared the three combinations NIVO + IPI, PEMBRO + AXI, and AVE + AXI in terms of PFS, OS, and ORR, with a trend in favor of the PEMBRO + AXI combination [18]. However, this meta-analysis did not include the last two combinations and was based solely on published, non-individual data. The IMDC consortium compared the NIVO + IPI or ICI (anti-PD-(L)1)-TKI combinations in 723 patients including 546 with I/P risk [19]. This retrospective analysis of a large number of patients required very careful interpretation as the quality of the data collected varied. The ORR was 37% vs. 59% in the NIVO + IPI and ICI-TKI arms, respectively, which was quite similar to the phase III data. In contrast, CR rates were similar in both arms, but lower than those in trials at only 4%. OS was not significantly different between the two types of treatment received: 40.2 vs. 39.7 months for NIVO + IPI vs. ICI-TKI (HR adjusted 0.92, *p* = 0.71), respectively. Based on the OS parameter alone, this analysis showed that there was no combination more effective than another in this poorly selected population. But it seemed that the benefit in OS was maintained over time for NIVO + IPI (constant HR), while the HR increased for the PEMBRO + AXI association. Finally, the meta-analysis of Quhal et al.—incorporating six studies (CheckMate-214, Keynote-426, IMmotion-151, JAVELIN Renal 101, Checkmate-9ER, and CLEAR), i.e., 5121 patients—suggested that ICI-TKI combinations provided superior PFS, ORR, and OS vs. ICI-ICI combinations, independent of the IMDC group [20]. Based on treatment classification analysis, NIVO + CABO was most likely to provide maximum OS (*p*-score 0.7573). These comparisons remain indirect and limited by the variability of patient characteristics in the trials evaluated (prognostic risk categories and PD-L1 expression) and differences in subsequent treatments received that may influence OS outcomes.

**Table 1:** Phase III trials of the immune checkpoint-based regimens evaluated in treatment-naïve mccRCC.

Parameter	CheckMate-214 [5,6]	JAVELIN Renal-101 [7,8]	KEYNOTE-426 [9,16]	CheckMate-9ER [11]	CLEAR [12]
Number of patients	1096	886	861	651	1069
Treatment arms	Nivolumab + ipilimumab (*n* = 550) vs. sunitinib (*n* = 546)	Avelumab + axitinib (*n* = 442) vs. sunitinib (*n* = 444)	Pembrolizumab + axitinib (*n* = 432) vs. sunitinib (*n* = 429)	Nivolumab + cabozantinib (*n* = 323) vs. sunitinib (*n* = 328)	Lenvatinib + pembrolizumab (*n* = 355) vs. lenvatinib + everolimus (*n* = 357) vs. sunitinib (*n* = 357)
Primary outcome	ORR, PFS and OS in I/P risk patients	PFS and OS in PD-L1+ patients	PFS and OS	PFS	PFS
Median follow-up, mo	55	13	42.8	18.1	27
Median OS, mo	ITT group: NR vs. 38.4 HR 0.69; 95% CI 0.59, 0.81 I/P risk: 48.1 vs. 26.6 HR 0.65; 95% CI 0.54, 0.78	PD-L1+: NE vs. 28.6 HR 0.83; 95% CI, 0.60, 1.15; *p* = 0.1301 ITT group: NE vs. NE HR 0.80; 95% CI, 0.62, 1.03 *p* = 0.0392	45.7 vs. 40 HR 0.73; 95% CI 0.60, 0.88; *p* < 0.001	NR vs. NR HR 0.60; 95% CI 0.40, 0.89; *p* = 0.0010	NR vs. NR vs. NR HR vs. Sun = 0.66; 95% CI 0.49, 0.69; *p* = 0.005
Median PFS, mo	ITT group: 12.2 vs. 12.3 HR 0.89; 95% CI 0.76, 1.05 I/P-risk: 11.2 vs. 8.3 HR 0.74; 95% CI 0.62, 0.88	PD-L1+: 13.8 vs. 7.0 HR 0.62; 95% CI 0.49, 0.78; *p* < 0.0001 ITT group: 13.3 vs. 8.0 HR 0.69; 95% CI, 0.57, 0.83; *p* < 0.0001	15.7 vs. 11.1 HR 0.68; 95% CI 0.58,0.80; *p* < 0.0001	16.6 vs. 8.3 HR 0.51; 95% CI 0.41, 0.64; *p* < 0.0001	23.9 vs. 14.7 vs. 9.2 HR vs. Sun: 0.39; 95% CI 0.32, 0.49; *p* < 0.001
ORR, %	ITT group: 39.1 vs. 32.4 I/P risk: 41.9 vs. 26.8	PD-L1+: 55.9 vs. 27.2 ITT group: 52.5 vs. 27.3	60.4 vs. 39.6	55.7 vs. 27.1	71.0 vs. 53.5 vs. 36.1
Complete response, %	ITT group: 10.7 vs. 2.6 I/P risk: 10.4 vs. 1.4	PD-L1+: 5.6 vs. 2.4 Overall pop: 3.8 vs. 2.0	10.0 vs. 3.5	8.0 vs. 4.6	16.1 vs. 9.8 vs. 4.2
Partial response, %	ITT group: 28.4 vs. 29.9 I/P risk: 31.5 vs. 25.4	PD-L1+: 50.4 vs. 24.8 Overall pop: 48.6 vs. 25.2	50.5 vs. 36.1	47.7 vs. 22.6	54.9 vs. 43.7 vs. 31.9
Stable disease, %	ITT group: 36.0 vs. 42.1 I/P risk: 30.8 vs. 44.3	PD-L1+: 27.0 vs. 41.4 Overall pop: 28.3 vs. 43.7	22.9 vs. 35.4	32.2 vs. 42.1	19.2 vs. 33.6 vs. 38.1
Progressive disease, %	ITT group: 17.6 vs. 14.1 I/P risk: 19.3 vs. 16.8	PD-L1+: 11.5 vs. 22.4 Overall pop: 12.4 vs. 19.4	11.3 vs. 17.0	5.6 vs. 13.7	5.4 vs. 7.3 vs. 14.0
Toxicities Events, %	All grades: 94.0 vs. 97.4 G3/4: 47.9 vs. 64.1	All grades: 99.5 vs. 99.3 G3/4: 71.2 vs. 71.5	All grades: 96.3 vs. 97.6 G3/5: 67.8 vs. 63.8	All grades: 100 vs. 99 G3/4: 75 vs. 71	All grades: 96.9 vs. 97.7 vs. 92.1 G3/4: 71.6 vs. 73.0 vs. 58.8

CI, confidence interval; HR, hazard ratio; I/P, intermediate/poor risk; ITT, intention-to-treat population; mccRCC, metastatic clear cell renal cell carcinoma; NE, not estimable; NR, not reached; ORR, objective response rate; OS, overall survival; PFS, progression-free survival.

### 3.2. IMDC Groups

The patient’s prognostic profile based on the IMDC risk score is a criterion that must be considered. The magnitude of the PFS benefit of the CABO + NIVO combination seemed particularly marked in patients with poor risk: HR 0.37 vs. 0.62 and 0.54 for patients with a good and intermediate risk, respectively. Similarly, OS benefits were greater in patients with poor risk, with a 63% reduction in the risk of death (HR 0.37 vs. 0.84 and 0.70 for a good and intermediate risk, respectively) [11]. The LENVA + PEMBRO combination presented similar results with a particularly significant OS benefit in the IMDC poor risk group (HR 0.30) and important response rates: 71% ORR and 16% CR. Given the significant percentage of CR, it may be an objective in its own right, but it remains to be seen whether it is influenced by the rather favorable population included in the trial or whether it is confirmed in real life or in other trials [12]. Moreover, the percentages of progression from the outset of both combinations were very low, at around 4–5% compared to 18% with NIVO + IPI [6,11,12]. Thus, in a patient at risk of rapid progression or presenting a threatening disease (e.g. threatening epiduritis with a high risk for spinal cord compression or bronchial compression) with a limited life expectancy, obtaining a rapid and important response could tip the decision towards an ICI-TKI combination (CABO + NIVO or LENVA + PEMBRO). Based on available data, it is still difficult to speculate whether the addition of CABO offers the combination a gain in efficacy on predominant or major bone lesions. Finally, an FDA analysis pooled individual data from 3447 patients from four randomized phase III trials of ICI-ICI (*n* = 1) or ICI-TKI (*n* = 3) combinations. Improvement in OS with combinations vs. SUN was found in I/P risk patients (HR 0.696; 95% CI: 0.62, 0.78) but not in patients with a good prognosis (HR 0.953; 95% CI: 0.72, 1.27) [21]. However, it should be noted that the monitoring, still too short in the trials, has not, for the time being, shown a benefit in OS or even PFS for the ICI-TKI combinations in these patients with a good prognosis, with only a benefit in ORR being found so far. In addition, IMDC favorable patients will be prone to receive first-line treatment for a long period of time, leading to an increased risk of experiencing cumulative TKI toxicities. Thus, in these patients, TKI is frequently interrupted which would be harmful, since it has been shown that their tumors are pro-angiogenic and highly sensitive to angiogenesis inhibitors [22]. As for ICI-ICI, PFS, and TR were lower than for TKI monotherapy, but the CR rates were higher and OS was comparable. According to the post-hoc analysis of the CheckMate 214 study performed according to the number of IMDC risk factors, a benefit of treatment with NIVO + IPI on SUN was found for all patients at intermediate risk, including those with one or two risk factors (ORR (40–44% vs. 16–38%), OS (HR 0.50–0.72), and PFS (HR 0.44–0.86)) [23]. All of these data favored combinations, including in patients with a good prognosis. Overall, it seemed relevant to have the second-line strategy in perspective when choosing the first-line treatment. Thus, in a patient without significant tumor volume and risk of rapid worsening, the criteria for the choice of treatment should include tolerance, continuation of treatment, and possible second-line treatment, leading the strategy towards ICI-ICI vs. ICI-TKI. Nevertheless, as part of a prolonged follow-up, the impact on the response rate—and possibly on OS—also leads us to consider an ICI-TKI combination. 

### 3.3. Potential Impact of PD-L1 Status

PD-L1 status is a recognized prognostic factor, but its predictive response value to ICI remains to be demonstrated [24,25]. The meta-analysis of Mori et al. [26] investigated the predictive value of PD-L1 expression in patients with mccRCC treated with first-line ICI combinations. Based on key clinical outcomes, including response rate and PFS, the authors found that PD-L1^+^ patients benefited more from ICI combinations than from SUN, with a PFS of 22 months vs. 6 months (HR 0.65, 95% CI: 0.57, 0.74, *p* < 0.001). In PD-L1^+^ patients, NIVO + IPI resulted in a more significant improvement in efficacy criteria compared with ICI-TKI for all IMDC risk groups. Examined study by study, in the I/P subgroup of the CheckMate-214 study [5], OS was significantly better in the NIVO + IPI arm compared to SUN regardless of PD-L1 status, although the magnitude of OS benefit was greater in the PD-L1^-^ subgroup (HR 0.73 vs. 0.45 for PD-L1^+^). For ICI-TKI, the data were heterogeneous [7,8,9,10,11]. In KEYNOTE-426, the superiority of PEMBRO + AXI over SUN was maintained regardless of PD-L1 status (HR 0.54 for PD-L1^+^ vs. HR 0.59 for PD-L1^₋^). Conversely, the CHECKMATE-9ER trial showed an impact of PD-L1 status, with a lower HR for OS in the PD-L1^-^ population (0.51 vs. 0.80 for PD-L1^+^). Similarly, in the CLEAR study, the OS benefit was particularly pronounced for PD-L1^-^ status (HR 0.50 vs. HR 0.76 for PD-L1^+^) [12]. However, these comparisons are questionable since the methods used to assess PD-L1 status differed according to the CheckMate-214/CheckMate-9ER, JAVELIN Renal 101 and KEYNOTE-426 studies: 28-8 clone (Dako), PD-L1 ≥ 1% in tumor cells, SP263 clone (Ventana), PD-L1 ≥ 1% in immune cells and 22C3 clone (Dako), Combined Positive Score (CPS) > 1% tumor cells plus immune cells, respectively [5,6,7,8,9,10,11]. This can probably partly explain why the proportion of the PD-L1^+^ population varied so widely from study to study. In addition, a biomarker analysis was performed using data from the CheckMate-214 study [27], in which the PD-L1 status was defined on tumor cells, but also according to the CPS combining tumor cells and immune cells. The recovered PD-L1^+^ level was 25% for tumor cells and 60% by CPS. The results of this analysis showed that when the proportion of positive patients increased, the OS benefit vs. SUN remained, but was of lower magnitude. Overall, the results diverged and harmonization of techniques in the future would allow a better comparison between the populations studied. To date, PD-L1 status does not seem to be a formal decision criterion in the choice of treatment, but it can be considered during the ICI-ICI vs. ICI-TKI decision. If PD-L1 status is assessed, it seems preferable to do so on tumor cells only since the most discriminant outcomes according to PD-L1 status has been shown in the CheckMate-214 study with ICI-ICI [27].

### 3.4. Tolerance Profile/Quality of Life

Tolerance and quality of life (QoL) are also important criteria for choosing the therapeutic strategy, especially as the potential lifespan increases. The type of adverse events (AEs) differs depending on the treatment or combination considered: there are more AEs with the ICI-TKI combination compared to ICI-ICI over the long term; however, when they occur in ICI-ICI, they may be more acute and unpredictable. Based on a meta-analysis that included four trials (CheckMate-214, Keynote-426, IMmotion-151 and JAVELIN Renal 101), ICI-based combinations were associated with a higher risk of all-grade pruritus (HR 3.11) and all-grade rash (HR 1.44) compared to patients treated with SUN. However, the combinations presented less grade 3/4 fatigue (HR 0.49) and nausea (HR 0.60) vs. SUN [28]. Another more recent meta-analysis incorporated the Checkmate-9ER and CLEAR studies [20]. Compared to the SUN, LENVA + PEMBRO was associated with the highest probability of treatment-related AEs of grade ≥3 (OR 1.84, 95% CI: 1.28, 2.64) and discontinuations (OR 3.55, 95% CI: 2.46, 5.12) [12]. NIVO + IPI was associated with the lowest rates of grade ≥3 AEs, but with a higher probability of endocrine-related AEs [20]. A higher probability of high-grade diarrhea was associated with PEMBRO + AXI and AVE + AXI. The duration of AEs was also different: in the CheckMate-214 study, ICI-ICI-related toxicity occurred mainly during the first four months of the study and subsequently stabilized while in the SUN arm, the rate of AEs remained more stable throughout the study, particularly for vascular, digestive, and hematological toxicities [5,6]. It should be noted that the benefit/risk balance of immunotherapy should be discussed in the first-line treatment for certain patient profiles, particularly those with inflammatory colitis, especially if they are active [29,30]. In patients over 75 years of age, OS was comparable but AEs were more frequent than in younger patients; however, this did not contraindicate the use of immunotherapy in these patients [6,31]. Given the small number of elderly patients enrolled in the trials, data from other or real-life trials remain necessary. 

In terms of QoL, patient-reported outcomes (PROs) were assessed as an exploratory criterion in the CheckMate-214 trial and showed that combined treatment resulted in fewer symptoms and a better QoL than with SUN [32]. In the Checkmate-9ER study, QoL was sustained over time with NIVO + CABO, while constant deterioration was observed with SUN. Combination therapy improved symptoms up to week 91 unlike SUN [11]. In an analysis of a secondary endpoint of HRQoL (Health-Related QoL) scores in the CLEAR trial, LENVA + PEMBRO demonstrated a similar time to first deterioration (TTD) in 14 out of 18 HRQoL and disease-related symptom scores, and a delay in TTD for physical functioning, dyspnea, appetite loss, and EQ-5D visual analog scale compared to SUN [33]. Overall, QoL improved when treated with ICI-ICI, but not with ICI-TKI due to continuous administration of antiangiogenics. It should be noted that in practice, induction of treatment with ICI-ICI requires close monitoring due to the specific nature of the AEs and access to a network of specialists and dedicated multidisciplinary consultative meetings (such as ImmunoTox).

### 3.5. Treatment Sequence: Second-Line and Subsequent Therapies

The therapeutic strategy is crucial for patients with a good prognosis: they have a life expectancy of several years and therefore a higher probability of receiving many lines unlike I/P patients. For the time being, there is no gain in OS, so it is too early to know whether a sequential approach with an antiangiogenic in the first-line treatment, based on the often-predominant angiogenic profile, and then immunotherapy in the second-line treatment, would really be inferior to a combination strategy from the outset. In patients with a good prognosis and a small-volume tumor for which a CR is achievable, the notion of the second-line treatment and the strategy of subsequent lines are important to consider. Based on data from the favorable prognostic patient group in the CheckMate-214 study [6], more than 50% of patients survived at 48 months (with an HR for OS of 0.69 in the NIVO + IPI arm vs. 0.65 in the SUN arm). However, there are numerous treatment options after a first-line treatment of SUN or post-NIVO + IPI, but fewer after CABO + NIVO or PEMBRO + LENVA. Indeed, after treatment with CABO, which has a strong anti-VEGFR2 effect, no solid data suggest the efficacy of SUN or AXI. It should be noted that HIF (Hypoxia Inducible Factor) inhibitors are being evaluated after these first-line strategies [34].

In patients with I/P risk who have received a first-line therapy with ICI-ICI combination, the question that arises is which TKI to choose for second-line therapy? A retrospective trial in 33 patients in the CheckMate 214 trial who received second-line TKI after ICI-ICI reported a median PFS of 8 months for first-generation TKI (sunitinib/pazopanib) and 7 months for second-generation TKI (axitinib/cabozantinib) (*p* = 0.66) [35]. This retrospective trial did not validate the feasibility of a second-line treatment by TKI after ICI-ICI or the choice of the first- or second-generation TKI molecule. Dudani et al. [36], using IMDC data, compared the efficacy of second-line treatment after ICI-ICI NIVO + IPI or after ICI-TKI. A total of 113 patients received ICI-TKI and 75 ICI-ICI in the first-line treatment, and 34 patients (30%) in the ICI-TKI group and 30 patients (40%) in the ICI-ICI group received a second-line treatment, mainly VEGF TKI (axitinib, cabozantinib, lenvatinib + everolimus, pazopanib and sunitinib). The second line response rate was 15% in the ICI-TKI group vs. 45% post-ICI-ICI (*p* = 0.04); however, the time to treatment failure (TTF) was not statistically different (3.7 vs. 5.4 months; *p* = 0.4). Updating of data in 142 patients, 103 of whom had received the second-line treatment, confirmed these results with a response rate that remained higher after ICI-ICI (37% vs. 12%, *p* < 0.01), but with no difference in OS or TTF [37]. Finally, a phase II study evaluating PEMBRO + LENVA after ICI, presented by Lee et al at ASCO 2020, reported an ORR of 47% in the 38 patients who received NIVO + IPI in first line [38]. The choice of TKI must therefore consider the patient’s profile and the fact that a proportion of patients will not reach a third-line treatment. However, the optimal sequence remains to be validated in the trials.

Another question is: Is the introduction of an anti-CTLA-4 in salvage therapy after a lack of response to an anti-PD-1 monotherapy (NIVO or PEMBRO) in the first-line treatment of interest? To date, the only data on the use of NIVO + IPI after prior anti-PD-(L)1 failure are based on four non-randomized phase II trials that were presented at the ESMO 2019 (TITAN-RCC) and ASCO 2020 (FRACTION-RCC, OMNIVORE, and HCRN GU16-260) congresses [39,40,41,42]. The pooled analysis of the four studies (*n* = 237 patients) confirmed a low response rate of 10.0% associated with 27.0% of grade ≥3 AEs [43]. Finally, a small retrospective study of 45 patients reported results of the combination of NIVO + IPI in second-line treatment post-anti-PD-1 alone or in combination and/or post-TKI: after a median follow-up of 12 months, the ORR was 20% and the median PFS was 4 months (0.8–19 months) [44]. Overall, the combination of NIVO (anti-PD-1) + IPI (anti-CTLA-4) in patients who have already received anti-PD-(L)1 treatment, but no anti-CTLA-4, did not seem an option to retain and supported administering anti-CTLA-4 only in the setting of first-line treatment.

## 4. Outlook

Beyond sequential therapeutic strategy trials, researching biomarkers predictive of response to ICI is also essential. Among the biomarkers studied is the PD-L1 status, but also the molecular profiling of the tumor. Thus, the BIONIKK study assessed personalized treatments with ICI alone or ICI-ICI or TKI according to tumor molecular characteristics in mccRCC [45]. Using an expression signature of 35 genes, patients were divided into four molecular groups (1 to 4). Patients in groups 1 and 4 were randomized to receive NIVO alone or NIVO + IPI (four administrations) followed by NIVO alone. Patients in groups 2 and 3 were randomized to receive either NIVO + IPI followed by NIVO alone or a TKI (sunitinib or pazopanib) according to the investigator. The study questioned the interest of establishing a routine tumor molecular profile to optimize the choice of treatment between immunotherapy monotherapy, or an ICI-ICI or ICI-TKI combination. First results presented at the 2019 ESMO meeting were encouraging [46].

Finally, other developments in the therapeutic arsenal are expected in the coming years with, on one hand, potential intensification with first-line triplet CABO + NIVO + IPI and, on the other, the introduction of anti-PD-1 in the adjuvant setting [47] which may increase survival but will also impact subsequent lines. Furthermore, the time to progression (within 6–12 months or more than 12 months after the end of anti-PD-1) will likely influence the choice. 

## 5. Conclusions

To conclude, currently the PEMBRO + AXI, CABO + NIVO (for all patients), and NIVO + IPI (for patients with I/P risk) combinations constitute the first-line management standard for mccRCC. However, multiplication of first-line treatment options continues and now no less than five combinations have robust data, with unfortunately no direct comparison study of the different combinations available. The choice of strategy must therefore be based on efficacy criteria, but also on the patient’s risk profile and tolerance to each treatment (Table 2), while keeping the options of the subsequent lines in perspective. Given the complexity of choice, therapeutic sequence data with second-line combinations will become essential to guide the therapeutic strategy. Even if these combinations were approved regardless of the tumor PD-L1 status, the use of predictive biomarkers of response to ICI could, in the future, help determine the best personalized treatment strategy for each patient.

## Figures and Tables

**Table 2:** Parameters guiding the choice of strategy between ICI/ICI and ICI/TKI combinations.

Parameter		ICI-ICI	ICI-TKI
Prolonged follow-up		✓	✓
Efficacy: overall	CR, OS ORR, PFS	✓ ✓	✓ ✓
Efficacy: subgroups	IMDC favorable IMDC intermediate/poor PD-L1+ PD-L1-	✘ ✓ ✓ ✓	✓ ✓ ✓ ✓
Tolerability	Overall Cardiovascular Immune-mediated	✓ ✓ ✘	✘ ✘ ✓
Quality of life		✓	✓
Subsequent line options		✓	✘

Green check mark: in favor; Orange check mark: lacks information or does not allow to conclude; Red cross: rather in disfavor; CR, complete response rate; ICI, immune checkpoint inhibitors; IMDC, International Metastatic RCC Database Consortium; ORR, objective response rate; OS, overall survival; PD-L1, Programmed cell death ligand 1; PFS, progression free-survival; TKI, tyrosine kinase inhibitors.

## References

[1] Lalani A.-K.A., McGregor B.A., Albiges L., Choueiri T.K., Motzer R., Powles T., Wood C., Bex A. (2019). Systemic Treatment of Metastatic Clear Cell Renal Cell Carcinoma in 2018: Current Paradigms, Use of Immunotherapy, and Future Directions. Eur. Urol..

[2] Salgia N.J., Dara Y., Bergerot P., Salgia M., Pal S.K. (2019). The Changing Landscape of Management of Metastatic Renal Cell Carcinoma: Current Treatment Options and Future Directions. Curr. Treat. Options Oncol..

[3] Pardoll D.M. (2012). The blockade of immune checkpoints in cancer immunotherapy. Nat. Rev. Cancer.

[4] Massari F., Rizzo A., Mollica V., Rosellini M., Marchetti A., Ardizzoni A., Santoni M. (2021). Immune-based combinations for the treatment of metastatic renal cell carcinoma: A meta-analysis of randomised clinical trials. Eur. J. Cancer.

[5] Motzer R.J., Tannir N.M., McDermott D.F., Arén Frontera O., Melichar B., Choueiri T.K., Plimack E.R., Barthélémy P., Porta C., George S. (2018). Nivolumab plus Ipilimumab versus Sunitinib in Advanced Renal-Cell Carcinoma. N. Engl. J. Med..

[6] Albiges L., Tannir N.M., Burotto M., McDermott D., Plimack E.R., Barthélémy P., Porta C., Powles T., Donskov F., George S. (2020). Nivolumab plus ipilimumab versus sunitinib for first-line treatment of advanced renal cell carcinoma: Extended 4-year follow-up of the phase III CheckMate 214 trial. ESMO Open.

[7] Motzer R.J., Penkov K., Haanen J., Rini B., Albiges L., Campbell M.T., Venugopal B., Kollmannsberger C., Negrier S., Uemura M. (2019). Avelumab plus Axitinib versus Sunitinib for Advanced Renal-Cell Carcinoma. N. Engl. J. Med..

[8] Choueiri T., Motzer R., Rini B., Haanen J., Campbell M., Venugopal B., Kollmannsberger C., Gravis-Mescam G., Uemura M., Lee J. (2020). Updated efficacy results from the JAVELIN Renal 101 trial: First-line avelumab plus axitinib versus sunitinib in patients with advanced renal cell carcinoma. Ann. Oncol..

[9] Rini B.I., Plimack E.R., Stus V., Gafanov R., Hawkins R., Nosov D., Pouliot F., Alekseev B., Soulières D., Melichar B. (2019). Pembrolizumab plus Axitinib versus Sunitinib for Advanced Renal-Cell Carcinoma. N. Engl. J. Med..

[10] Powles T., Plimack E.R., Soulières D., Waddell T., Stus V., Gafanov R., Nosov D., Pouliot F., Melichar B., Vynnychenko I. (2020). Pembrolizumab plus axitinib versus sunitinib monotherapy as first-line treatment of advanced renal cell carcinoma (KEYNOTE-426): Extended follow-up from a randomised, open-label, phase 3 trial. Lancet Oncol..

[11] Choueiri T.K., Powles T., Burotto M., Escudier B., Bourlon M.T., Zurawski B., Oyervides-Juárez V.M., Hsieh J.J., Basso U., Shah A.Y. (2021). Nivolumab plus Cabozantinib versus Sunitinib for Advanced Renal-Cell Carcinoma. N. Engl. J. Med..

[12] Motzer R.J., Porta C., Eto M., Powles T., Grünwald V., Hutson T.E., Alekseev B., Rha S.Y., Kopyltsov E., Méndez-Vidal M.J. (2021). Phase 3 trial of lenvatinib (LEN) plus pembrolizumab (PEMBRO) or everolimus (EVE) versus sunitinib (SUN) monotherapy as a first-line treatment for patients (pts) with advanced renal cell carcinoma (RCC) (CLEAR study). JCO.

[13] Escudier B., Porta C., Schmidinger M., Rioux-Leclercq N., Bex A., Khoo V., Grünwald V., Gillessen S., Horwich A., ESMO Guidelines Committee (2019). Renal Cell Carcinoma: ESMO Clinical Practice Guidelines for Diagnosis, Treatment and Follow-Up. Ann. Oncol..

[14] Powles T., Albiges L., Bex A., Grünwald V., Porta C., Procopio G., Schmidinger M., Suárez C., de Velasco G., ESMO Guidelines Committee (2021). ESMO Clinical Practice Guideline Update on the Use of Immunotherapy in Early Stage and Advanced Renal Cell Carcinoma. Ann. Oncol..

[15] Aeppli S., Schmaus M., Eisen T., Escudier B., Grünwald V., Larkin J., McDermott D., Oldenburg J., Porta C., Rini B.I. (2021). First-line treatment of metastatic clear cell renal cell carcinoma: A decision-making analysis among experts. ESMO Open.

[16] Rini B.I., Plimack E.R., Stus V., Waddell T., Gafanov R., Pouliot F., Nosov D., Melichar B., Soulieres D., Borchiellini D. (2021). Pembrolizumab (Pembro) plus Axitinib (Axi) versus Sunitinib as First-Line Therapy for Advanced Clear Cell Renal Cell Carcinoma (CcRCC): Results from 42-Month Follow-up of KEYNOTE. JCO.

[17] Haanen J.B.A.G., Larkin J., Choueiri T.K., Albiges L., Rini B.I., Atkins M.B., Schmidinger M., Penkov K., Thomaidou D., Wang J. (2021). Efficacy of Avelumab + Axitinib (A + Ax) versus Sunitinib (S) by IMDC Risk Group in Advanced Renal Cell Carcinoma (ARCC): Extended Follow-up Results from JAVELIN Renal. JCO.

[18] ElAidi R., Phan L., Borchiellini D., Barthelemy P., Ravaud A., Oudard S., Vano Y. (2020). Comparative Efficacy of First-Line Immune-Based Combination Therapies in Metastatic Renal Cell Carcinoma: A Systematic Review and Network Meta-Analysis. Cancers.

[19] Gan C.L., Dudani S., Wells J.C., Schmidt A.L., Bakouny Z., Szabados B., Parnis F., Wong S., Lee J.-L., de Velasco G. (2021). Outcomes of first-line (1L) immuno-oncology (IO) combination therapies in metastatic renal cell carcinoma (mRCC): Results from the International mRCC Database Consortium (IMDC). JCO.

[20] Quhal F., Mori K., Remzi M., Fajkovic H., Shariat S.F., Schmidinger M. (2021). Adverse events of systemic immune-based combination therapies in the first-line treatment of patients with metastatic renal cell carcinoma: Systematic review and network meta-analysis. Curr. Opin. Urol..

[21] Lee D., Gittleman H., Weinstock C., Suzman D.L., Bloomquist E., Agrawal S., Brave M.H., Brewer J.R., Singh H., Tang S. (2021). An FDA-pooled analysis of frontline combination treatment benefits by risk groups in metastatic renal cell carcinoma (mRCC). J. Clin. Oncol..

[22] Verbiest A., Renders I., Caruso S., Couchy G., Job S., Laenen A., Verkarre V., Rioux-Leclercq N., Schöffski P., Vano Y.A. (2019). Clear-cell Renal Cell Carcinoma: Molecular Characterization of IMDC Risk Groups and Sarcomatoid Tumors. Clin. Genitourin. Cancer.

[23] Escudier B., Motzer R.J., Tannir N.M., Porta C., Tomita Y., Maurer M.A., McHenry M.B., Rini B.I. (2020). Efficacy of Nivolumab plus Ipilimumab According to Number of IMDC Risk Factors in CheckMate. Eur. Urol..

[24] Flaifel A., Xie W., Braun D.A., Ficial M., Bakouny Z., Nassar A.H., Jennings R.B., Escudier B., George D.J., Motzer R.J. (2019). PD-L1 Expression and Clinical Outcomes to Cabozantinib, Everolimus, and Sunitinib in Patients with Metastatic Renal Cell Carcinoma: Analysis of the Randomized Clinical Trials METEOR and CABOSUN. Clin. Cancer Res..

[25] Iacovelli R., Nolè F., Verri E., Renne G., Paglino C., Santoni M., Cossu Rocca M., Giglione P., Aurilio G., Cullurà D. (2016). Prognostic Role of PD-L1 Expression in Renal Cell Carcinoma. A Systematic Review and Meta-Analysis. Target. Oncol..

[26] Mori K., Abufaraj M., Mostafaei H., Quhal F., Fajkovic H., Remzi M., Karakiewicz P.I., Egawa S., Schmidinger M., Shariat S.F. (2021). The Predictive Value of Programmed Death Ligand 1 in Patients with Metastatic Renal Cell Carcinoma Treated with Immune-checkpoint Inhibitors: A Systematic Review and Meta-analysis. Eur. Urol..

[27] Motzer R.J., Choueiri T.K., McDermott D.F., Powles T., Yao J., Ammar R., Papillon-Cavanagh S., Saggi S.S., McHenry B.M., Ross-Macdonald P. (2020). Biomarker analyses from the phase III CheckMate 214 trial of nivolumab plus ipilimumab (N+I) or sunitinib (S) in advanced renal cell carcinoma (aRCC). JCO.

[28] Massari F., Mollica V., Rizzo A., Cosmai L., Rizzo M., Porta C. (2020). Safety evaluation of immune-based combinations in patients with advanced renal cell carcinoma: A systematic review and meta-analysis. Expert Opin. Drug Saf..

[29] Abu-Sbeih H., Faleck D.M., Ricciuti B., Mendelsohn R.B., Naqash A.R., Cohen J.V., Sellers M.C., Balaji A., Ben-Betzalel G., Hajir I. (2020). Immune Checkpoint Inhibitor Therapy in Patients With Preexisting Inflammatory Bowel Disease. J. Clin. Oncol..

[30] Haanen J., Ernstoff M., Wang Y., Menzies A., Puzanov I., Grivas P., Larkin J., Peters S., Thompson J., Obeid M. (2020). Autoimmune diseases and immune-checkpoint inhibitors for cancer therapy: Review of the literature and personalized risk-based prevention strategy. Ann. Oncol..

[31] Araujo D.V., Wells J.C., Hansen A.R., Dizman N., Pal S.K., Beuselinck B., Donskov F., Gan C.L., Yan F., Tran B. (2021). Efficacy of immune-checkpoint inhibitors (ICI) in the treatment of older adults with metastatic renal cell carcinoma (mRCC) – an International mRCC Database Consortium (IMDC) analysis. J. Geriatr. Oncol..

[32] Cella D., Grünwald V., Escudier B., Hammers H.J., George S., Nathan P., Grimm M.-O., Rini B.I., Doan J., Ivanescu C. (2019). Patient-reported outcomes of patients with advanced renal cell carcinoma treated with nivolumab plus ipilimumab versus sunitinib (CheckMate 214): A randomised, phase 3 trial. Lancet Oncol..

[33] Motzer R.J., Porta C., Alekseev B., Rha S.Y., Choueiri T.K., Mendez-Vidal M.J., Hong S.-H., Kapoor A., Goh J.C., Eto M. (2021). Health-related quality-of-life (HRQoL) analysis from the phase 3 CLEAR trial of lenvatinib (LEN) plus pembrolizumab (PEMBRO) or everolimus (EVE) versus sunitinib (SUN) for patients (pts) with advanced renal cell carcinoma (aRCC). JCO.

[34] Choueiri T.K., Bauer T.M., Papadopoulos K.P., Plimack E.R., Merchan J.R., McDermott D.F., Michaelson M.D., Appleman L.J., Thamake S., Perini R.F. (2021). Inhibition of hypoxia-inducible factor-2α in renal cell carcinoma with belzutifan: A phase 1 trial and biomarker analysis. Nat. Med..

[35] Auvray M., Auclin E., Barthelemy P., Bono P., Kellokumpu-Lehtinen P., Gross-Goupil M., De Velasco G., Powles T., Mouillet G., Vano Y.A. (2019). Second-line targeted therapies after nivolumab-ipilimumab failure in metastatic renal cell carcinoma. Eur. J. Cancer.

[36] Dudani S., Graham J., Wells J.C., Bakouny Z., Pal S.K., Dizman N., Donskov F., Porta C., de Velasco G., Hansen A. (2019). First-line Immuno-Oncology Combination Therapies in Metastatic Renal-cell Carcinoma: Results from the International Metastatic Renal-cell Carcinoma Database Consortium. Eur. Urol..

[37] Stukalin I., Dudani S., Wells C., Gan C.L., Pal S.K., Dizman N., Powles T., Donskov F., Wood L., Bakouny Z. (2020). Second-line VEGF TKI after IO combination therapy: Results from the International Metastatic Renal Cell Carcinoma Database Consortium (IMDC). JCO.

[38] Lee C.-H., Shah A.Y., Hsieh J.J., Rao A., Pinto A., Bilen M.A., Cohn A.L., Di Simone C., Shaffer D.R., Sarrio R.G. (2020). Phase II trial of lenvatinib (LEN) plus pembrolizumab (PEMBRO) for disease progression after PD-1/PD-L1 immune checkpoint inhibitor (ICI) in metastatic clear cell renal cell carcinoma (mccRCC). JCO.

[39] Grimm M.-O., Schmidinger M., Martinez I.D., Schinzari G., Esteban E., Schmitz M., Schumacher U., Baretton G., Barthelemy P., Melichar B. (2019). Tailored immunotherapy approach with nivolumab in advanced renal cell carcinoma (TITAN-RCC). Ann. Oncol..

[40] Atkins M.B., Jegede O., Haas N.B., McDermott D.F., Bilen M.A., Drake C.G., Sosman J.A., Alter R.S., Plimack E.R., Rini B.I. (2020). Phase II study of nivolumab and salvage nivolumab + ipilimumab in treatment-naïve patients (pts) with advanced renal cell carcinoma (RCC) (HCRN GU16-260). J. Clin. Oncol..

[41] McKay R.R., McGregor B.A., Xie W., Braun D.A., Wei X., Kyriakopoulos C.E., Zakharia Y., Maughan B.L., Rose T.L., Stadler W.M. (2020). Optimized Management of Nivolumab and Ipilimumab in Advanced Renal Cell Carcinoma: A Response-Based Phase II Study (OMNIVORE). J. Clin. Oncol..

[42] Choueiri T.K., Kluger H.M., George S., Tykodi S.S., Kuzel T.M., Perets R., Nair S., Procopio G., Carducci M.A., Castonguay V. (2020). FRACTION-RCC: Innovative, high-throughput assessment of nivolumab + ipilimumab for treatment-refractory advanced renal cell carcinoma (aRCC). JCO.

[43] Carril-Ajuria L., Lora D., Carretero-González A., Martín-Soberón M., Rioja-Viera P., Castellano D., De Velasco G. (2021). Systemic Analysis and Review of Nivolumab-ipilimumab Combination as a Rescue Strategy for Renal Cell Carcinoma After Treatment With Anti-PD-1/PD-L1 Therapy. Clin. Genitourin. Cancer.

[44] Gul A., Stewart T.F., Mantia C.M., Shah N.J., Gatof E.S., Long Y., Allman K.D., Ornstein M.C., Hammers H.J., McDermott D.F. (2020). Salvage Ipilimumab and Nivolumab in Patients With Metastatic Renal Cell Carcinoma After Prior Immune Checkpoint Inhibitors. J. Clin. Oncol..

[45] Epaillard N., Simonaggio A., Elaidi R., Azzouz F., Braychenko E., Thibault C., Sun C.-M., Moreira M., Oudard S., Vano Y.-A. (2020). BIONIKK: A phase 2 biomarker driven trial with nivolumab and ipilimumab or VEGFR tyrosine kinase inhibitor (TKI) in naïve metastatic kidney cancer. Bull. Cancer.

[46] Vano Y., ElAidi R., Bennamoun M., Chevreau C., Borchiellini D., Pannier D., Maillet D., Gross-Goupil M., Tournigand C., Laguerre B. (2020). LBA25 Results from the phase II biomarker driven trial with nivolumab (N) and ipilimumab or VEGFR tyrosine kinase inhibitor (TKI) in naïve metastatic kidney cancer (m-ccRCC) patients (pts): The BIONIKK trial. Ann. Oncol..

[47] Choueiri T.K., Tomczak P., Park S.H., Venugopal B., Ferguson T., Chang Y.-H., Hajek J., Symeonides S.N., Lee J.L., Sarwar N. (2021). Adjuvant Pembrolizumab after Nephrectomy in Renal-Cell Carcinoma. N. Engl. J. Med..

